# Staphylococcal Superantigens Spark Host-Mediated Danger Signals

**DOI:** 10.3389/fimmu.2016.00023

**Published:** 2016-02-02

**Authors:** Teresa Krakauer, Kisha Pradhan, Bradley G. Stiles

**Affiliations:** ^1^Department of Immunology, Molecular Translational Sciences Division, United States Army Medical Research Institute of Infectious Diseases, Fort Detrick, Frederick, MD, USA; ^2^Biology Department, Wilson College, Chambersburg, PA, USA

**Keywords:** SEB, superantigens, toxic shock, damage response, therapy, animal models

## Abstract

Staphylococcal enterotoxin B (SEB) of *Staphylococcus aureus*, and related superantigenic toxins produced by myriad microbes, are potent stimulators of the immune system causing a variety of human diseases from transient food poisoning to lethal toxic shock. These protein toxins bind directly to specific Vβ regions of T-cell receptors (TCR) and major histocompatibility complex (MHC) class II on antigen-presenting cells, resulting in hyperactivation of T lymphocytes and monocytes/macrophages. Activated host cells produce excessive amounts of proinflammatory cytokines and chemokines, especially tumor necrosis factor α, interleukin 1 (IL-1), IL-2, interferon γ (IFNγ), and macrophage chemoattractant protein 1 causing clinical symptoms of fever, hypotension, and shock. Because of superantigen-induced T cells skewed toward TH1 helper cells, and the induction of proinflammatory cytokines, superantigens can exacerbate autoimmune diseases. Upon TCR/MHC ligation, pathways induced by superantigens include the mitogen-activated protein kinase cascades and cytokine receptor signaling, resulting in activation of NFκB and the phosphoinositide 3-kinase/mammalian target of rapamycin pathways. Various mouse models exist to study SEB-induced shock including those with potentiating agents, transgenic mice and an “SEB-only” model. However, therapeutics to treat toxic shock remain elusive as host response genes central to pathogenesis of superantigens have only been identified recently. Gene profiling of a murine model for SEB-induced shock reveals novel molecules upregulated in multiple organs not previously associated with SEB-induced responses. The pivotal genes include intracellular DNA/RNA sensors, apoptosis/DNA damage-related molecules, immunoproteasome components, as well as antiviral and IFN-stimulated genes. The host-wide induction of these, and other, antimicrobial defense genes provide evidence that SEB elicits danger signals resulting in multi-organ damage and toxic shock. Ultimately, these discoveries might lead to novel therapeutics for various superantigen-based diseases.

## The Bacterium: *Staphylococcus aureus*

*Staphylococcus aureus* causes many human and animal diseases, some of them are life-threatening and found throughout the world (1–4). Humans are naturally colonized by *S. aureus* that often cause no problems and are considered a “harmless” commensal. However, when given an opportunity (i.e., weakened immune system, compromised epidermis, or mucosa, etc.), this bacterium can cause various diseases that become life threatening.

*Staphylococcus aureus* is a Gram-positive coccus and member of the Micrococcaceae family, which includes non-pathogenic genera found in soil, water, and on skin. Both planktonic- and biofilm-based versions of *S. aureus* can cause disease in an infected host, subsequently becoming (at times) quite difficult to clear from the body (5, 6). Characteristics of this genus called *Staphylococcus* (“staphyle” in Greek, meaning grape cluster and “coccus” meaning grain or berry) and species called *aureus* (“golden” in Latin, as per colony color on agar media), include Gram-positive, non-spore forming, facultative, β-hemolytic, catalase-positive, mannitol fermentation, and salt tolerant (7).

*Staphylococcus aureus* is known for producing many different protein toxins involved in pathogenesis. Clearly, the bacterium is very good at surviving harsh conditions in/on a host by using a vast array of virulence factors that promote the many diseases caused by infiltrating *S. aureus* and their toxins. One group of toxins, the staphylococcal enterotoxins (SEs), is the focus of this review. Antibiotic-resistant strains (community-, nursing home-, and hospital-acquired) are a particular problem in health care and its associated economics, requiring effective infection control plans that prevent *S. aureus* spread locally and globally (8).

*Staphylococcus aureus* was first described in the early 1880s by a Scotsman, Dr. Alexander Ogston, after microscopy analysis of over a hundred, pus-filled abscesses of human origins (9). *S. aureus* readily colonizes mammalian epidermis/dermis, mucosa, soft tissues, bone, and medical devices such as catheters. Furthermore, medically relevant strains of the bacterium have become variably resistant to many antibiotics, including methicillin and vancomycin, which negatively impacts psychological and economic aspects of human society (10–13). *S. aureus* strains that are methicillin resistant (MRSA) are commonly treated first with vancomycin. Both methicillin and vancomycin target bacterial cell-wall synthesis. Other antimicrobials that have been used for fighting *S. aureus* include (1) linezolid (inhibits protein synthesis at the 50S ribosome); (2) daptomycin (inserts into membranes); (3) fusidic acid (inhibits protein elongation at the ribosome); (4) teicoplanin (like vancomycin, inhibits cell wall synthesis but not used in the United States); and (5) tigecycline (inhibits protein synthesis at the 30S ribosome) (14, 15).

For various reasons, alternative methods for treating ­antibiotic-resistant strains of *S. aureus* (i.e., MRSA) are being explored by different groups around the world (16–23). Some of the exotic sources for these novel drugs include snake venom, tree bark/stems/leaves, and carnivorous plants. Risk factors for MRSA colonization include antibiotic exposure, admittance into an intensive care unit, surgery, long-term care residency, and exposure to others carrying MRSA (4, 24). In 2011, the United States Centers for Disease Control and Prevention reported >80,000 MRSA cases that were life threatening, with >11,000 fatalities (14%) (22, 25). This is remarkably a number of deaths greater than that attributed to acquired immunodeficiency syndrome (AIDS) for the same time period. Furthermore, the economic burden of MRSA in the United States is upwards to $13.8 billion/year and represents one of the most costly, acute infectious diseases requiring treatment (10). Invasive forms of MRSA include the following in order of prevalence: bacteremia, pneumonia, cellulitis, osteomyelitis, endocarditis, and septic shock (24). Black males older than 65 years are the most common patients suffering from invasive MRSA; however, the reasons for this are to date unknown (24). MRSA from hospital/health-care settings are perhaps the origins of community strains now seen throughout the human population (4). The latter types of *S. aureus* are often more susceptible to antibiotics than those from hospital/health-care settings.

From a historical perspective, MRSA was detected within a year after introducing this new beta-lactam antibiotic called methicillin in 1959, further highlighting the rapid adaptability of this microbe (26, 27). Methicillin was meant to overcome the increasingly prevalent, penicillin-resistant strains of *S. aureus* first detected during the mid-1940s in hospitals and throughout the community. These strains were isolated just 2 years after penicillin’s introduction into clinical practice (27). This pattern of antibiotic resistance is evident with various pathogens, suggesting furthermore the importance of developing truly novel antimicrobials and embracing a paradigm shift for discovering/creating antimicrobials. Perhaps vaccines will play a bigger role in fighting various pathogens, in the future?

Transmission of antibiotic-resistant strains between livestock and humans working with and/or consuming the former is a real problem, poorly understood to date (28–31). Furthermore, aerosol transmission of MRSA from farm animals or human patients in hospital wards, to humans, is also a scary reality (32–34). As just one known example of *S. aureus* transmission between species, strain CC398 was most commonly associated with asymptomatic colonization of swine, but now causes invasive infections of humans (35). Evidently, farm animals are an important reservoir for disease-causing *S. aureus* that colonize humans. Humans (~30%) can be asymptomatic carriers of *S. aureus* strains that possess virulence factor genes encoding antibiotic resistance, toxins, and other proteins that promote some nasty, potentially fatal diseases (1, 3, 36–40) (Table 1). The risk factors are many for children carrying MRSA nasally, and include (1) <4 years of age; (2) being male; (3) family size >4; (4) antibiotic use during previous 3 months; (5) smoking by parents; and (6) sleeping with parents (39). For adults, risk factors that enhance carriage of *S. aureus* include: (1) being male; (2) having diabetes; (3) use of hormonal contraception by women; (4) close physical contact with others (i.e., sports); and interestingly (5) being a non-smoker (40).

**Table 1:** ***Staphylococcus aureus*: some virulence factors and diseases**.

Virulence factors
Antibiotic resistance (multiple mechanisms)
Biofilm/capsule
Coagulase
Exfoliative toxin
Microbial surface components recognizing adhesive matrix molecules (MSCRAMMs)
Plasminogen activator
Pore-forming toxins (hemolysins and leukocidins)
Quorum sensing mechanism
Superantigens (enterotoxins and toxic shock syndrome toxin-1)
Toll/interleukin 1 receptor (TIR)-like protein
**Diseases**
Bacteremia
Endocarditis
Osteomyelitis
Pneumonia
Pyoarthrosis
Skin and soft tissue infections (boils, cellulitis, impetigo, scalded-skin syndrome, sty)
Toxic shock syndrome

Clearly, *Homo sapiens* are not strangers to hosting *S. aureus* on, and in, themselves. This bacterium is rather adaptable and employs various virulence factors seemingly important in its survival. Are we, in the near and distant future, able to co-adapt and effectively counter this microbial medical challenge, which is just one of many? We now focus upon one remarkable virulence factor, a family of protein toxins that vary in structure and biological effects upon mammals: the SEs.

## The Staphylococcal Enterotoxins

From a historical perspective, the SEs (and *S. aureus*) were first described as “ptomaine” (derived from Greek “ptoma,” meaning corpse) in 1914 by Barber (41), after human consumption of cow’s milk contaminated by staphylococci (from the cow) led to vomiting and diarrhea of unsuspecting visitors. Family members were remarkably resistant to this poisoning effect, perhaps due to repeated exposure and development of toxin-specific immunity. Barber included himself as a “human guinea pig” to prove his point that milk from this one farm, during the hot season in the Philippines, was indeed the medium for enteric illness. His final analysis revealed that short-term storage of the milk at ambient temperature enabled staphylococcal growth and toxin production (41).

Dack et al. reported in 1930 an enteric-acting toxin produced by a “yellow hemolytic *Staphylococcus*” isolated from sponge cake involved in food poisoning (42). Upon administering culture filtrate of the isolated bacterium grown in the laboratory to a rabbit (intravenous) or humans (*n* = 3; oral), there was respectively death and uniform nausea/diarrhea. Some of the SEs were first purified by various groups during the 1950s (43–45), further linked to a major cause of food poisoning evident around the world, and found to stimulate specific subsets of T cells leading to shock (1, 46, 47).

Regarding staphylococcal food poisoning and diagnosis, the latter can be done upon suspected food/drink using classic microbiology and agar plates (10^5^ viable *S. aureus*/gram food), or by direct detection of toxin (47). SE detection in food is most commonly accomplished by employing immunological techniques (i.e., commercially available enzyme/fluorescent immune assays) that are to date limited in detecting relatively few toxin types. However, next generation assays involving mass spectrometry are now being developed for the future pending higher throughput and cost-effectiveness (47).

The SEs are characteristically stable proteins resistant to high heat, low pH, freezing, drying, and proteases. Partly because of their stable nature and powerful effects upon humans at very low doses, the SEs (particularly SEB) have been studied from a biodefense perspective spanning decades of research by various countries (48, 49). SEB is also considered a Category B select agent by various United States federal agencies. SEB, when inhaled, can induce several symptoms within 120 min involving an aching feeling (head and muscles), increased heartbeat, coughing, enteric dysfunction (i.e., nausea, vomiting, and diarrhea), as well as eye irritation (49). Nanogram levels of inhaled SEB are incapacitating while microgram levels can be fatal. SEB adversely affects the immune system, and it is plausible that opportunistic viruses and bacteria can cause further harm to the host exposed to any SE (50–52).

Upon ingestion, the SEs (A–V and counting) are responsible for a prevalent form of food poisoning globally (1, 38, 46, 47, 53). There are five, sequence-based groups of SEs generally recognized to date (1). These single-chain proteins (~25 kDa) are generally composed of two domains containing both alpha helices and beta sheets, separated by a groove, as evidenced by one of the first SEs to be crystallized: SEB (54). Since the mid-2000s, the literature has also adopted the designation of SE-like (SEl) (55). This defines a staphylococcal protein that shares amino acid sequence homology with a previously characterized SE, yet has not to date been tested (or at least reported in the literature) for enteric effects in primates (56). Regarding staphylococcal food poisoning, SEA is the most commonly detected culprit in the United States, followed by SED and SEB. Classic poisoning due to food-based SEs can occur after ingesting processed meats or dairy products contaminated by improper handling (i.e., *S. aureus* transfer from the skin of a food handler to food) and subsequent storage of food at an elevated temperature conducive to bacterial growth. Depending upon the *S. aureus* strain, there can be one (or more) SEs released into the tainted food. Following consumption of SEs in food/drink, one may still experience a general malaise 24–72 h later (56). Poisoning by the SEs *via* many different food types is rarely fatal for healthy individuals, and occurs around the world; however, as is often the case with many diseases, the very young and old represent higher risk groups for severe morbidity and possible death due to SE exposure (57).

Exactly how the SEs cause enteric illness is still remarkably unresolved, but prostaglandins and leukotrienes might mediate the effects (58, 59). Serotonin release in the intestines and vagal nerve stimulation may also be involved in SE-induced emesis (60, 61). The mode of action is seemingly more complex than many other enterotoxins that work directly upon gastric epithelial cells and/or supporting matrix.

The term “superantigen” commonly describes the *S. aureus* SEs and toxic shock syndrome toxin-1 (TSST-1), while related streptococcal pyrogenic exotoxins (SPEs), also known as superantigens, are produced by *Streptococcus pyogenes*. Superantigen was first used in late 1980s literature to describe microbial proteins that activate a large population of specific T-cells at very minute (picogram) concentrations (62, 63). Typical “conventional” antigens stimulate far fewer T-cells at higher concentrations. Superantigens and conventional counterparts also differ by: (1) superantigens bind to the exterior of the peptide-binding groove of major histocompatibility complex class II (MHC II); (2) superantigens bind to different MHC II types; (3) superantigenic effects occur without internalization and antigen processing; and (4) T-cell receptor (TCR) recognition of a superantigen: MHC II complex requires the variable region of a TCR β chain (Vβ), not the Vα–Vβ chain used by conventional antigens (62–67). From a virulence perspective, superantigens corrupt host immunity that then enables pathogen survival.

Microbial superantigens are reportedly produced by other bacterial genera (*Mycobacterium*, *Mycoplasma*, *Yersinia*), fungi (*Candida*), and even viruses (herpes, rabies), thus suggesting a conserved and successful strategy employed throughout Nature (68). Versus superantigens of *Staphylococcus* and *Streptococcus*, those from the other aforementioned microbes are poorly characterized to date.

## Receptor Binding and Signal Transduction

Superantigens interact with both CD4 and CD8 T-cells and the signaling, post-TCR binding, is similar to conventional antigen binding to TCR; however, binding is to specific Vβ regions of TCR unlike conventional antigens. In addition, the interaction of superantigen with MHC class II on antigen-presenting cells (APC) is also different, with superantigens binding outside the peptide-binding groove of MHC class II. Structural and binding studies indicate at least two different binding sites on MHC class II for SE and TSST-1. A common overlapping binding region exists on HLA-DR for these toxins, referred to as the generic MHC class II binding site involving the α chain. An additional binding site with higher affinity can be found within the C-terminus of SEA/SED/SEH, which binds to the HLA-DR β chain in a Zn^2+^-dependent manner (69–71). The presence of two MHC binding sites allows SEA to cross-link MHC class II on APC and activate monocytes, inducing potent proinflammatory mediators. The binding of staphylococcal superantigens to MHC class II, and bridging to TCR, activates a high percentage of T cells. Two decades of intense investigations focused upon superantigen binding to cellular receptors reveal how superantigens activate cells of the immune system (46, 62, 72–76). Interaction of superantigen with TCR transmits the classical first signal for T-cell activation. The binding of superantigen to costimulatory receptors such as CD28 promotes supramolecular clusters, stabilizes cellular interaction, and optimizes protein kinase signal transduction (77). CD28 co-stimulation enhances mRNA stability of IL-2 and T-cell survival by increased expression of anti-apoptotic Bcl-xl (78). Other cell-surface molecules such as CD2, CD11a/ICAM-1, and ELAM facilitate optimal activation of endothelial and T cells by SEB (79). TCR and costimulatory receptors activate protein tyrosine kinases (PTKs), LCK and ZAP-70, resulting in phospholipase C gamma (PLCγ) activation, the release of intracellular second messengers, and subsequent protein kinase C (PKC) activation (67, 80, 81). Accompanying this T-cell activation is F-actin polymerization and increased intracellular Ca^2+^. PTK and PKC activation lead to mitogen-activated protein kinase (MAPK), extracellular signal regulated kinase (ERK), and cJun N-terminal kinase (JNK) pathways that activate transcriptional factors NFκB, NF-AT, and AP-1 (67, 81, 82). PKCθ activation leads to CARMA1, Bcl10, and MALT1 (CBM) complexes that induce NFκB transcriptional activation and controls T-cell proliferation (83). Many proinflammatory cytokine genes contain NFκB-binding sites in their promotor region, and are induced by NFκB (84). The NFκB cascade is a major signal transduction pathway for many pattern recognition receptors (PRR), such as toll-like (TLR) and proinflammatory cytokine receptors (85). The cytokines IL-1, TNFα, IFNγ, IL-2, IL-6 and chemokines, specifically MCP-1, are induced by superantigens (74, 75, 86). IL-1 and TNFα also activate fibroblasts, epithelial and endothelial cells to induce NFκB and additional mediators, cell adhesion molecules, and tissue proteases (87). Mediators produced by superantigen-activated cells exert profound damaging effects on the immune and cardiovascular system, culminating in multi-organ dysfunction and lethal shock. PTKs and T-cell cytokines also activate phosphoinositide 3 kinase (PI3K), signaling protein kinase B (Akt), and mammalian target of rapamycin complex 1 (mTORC1) downstream (88). Two signaling molecules, NFκB and mTORC1, are key hubs mediating the major biological responses to SE from TCR, co-stimulator CD28, and cytokine signaling.

## PI3K/mTORC1 Pathway in T-Cell Activation

T-cell activation *via* the TCR-CD3 complex subsequently activates membrane proximal PTKs that phosphorylate TCR intracellular components, other cellular substrates, as well as adaptors (67, 80–82). PLCγ cleaves phosphatidylinositol 4,5-bisphosphate, generating second messengers to activate PKC and the proto-oncogene Ras. PTK also activates PI3K, generating several inositol phospholipids and ultimately activating Akt and PKCθ (89). A number of receptors besides TCR, including those for CD28, IL-2 (IL-2R), insulin, growth factor, and the G-protein-coupled receptor (GPCR), also transduce activation signals upon ligand binding *via* the PI3K pathway. The cytosolic signalsome formed by PKCθ and CBM is located at an immunological synapse formed after T-cell activation by anti-CD3 and -CD28 (89, 90). CBM complexes activate the inhibitor of κB (IκB) kinase complex (IKK) through ubiquitin ligases (91). IKK phosphorylates IκB, releasing NF-κB for nuclear translocation and gene activation. Nuclear NFκB binds DNA coding for proinflammatory cytokines and many other NFκB target genes, activating diverse proinflammatory mediators, as well as pro- and anti-apoptotic molecules.

Toxic shock syndrome toxin-1 induces inositol phospholipid turnover, PKC translocation, and calcium mobilization in human T-cells resembling responses from those of a mitogenic signal (92). SEE uses an alternative LCK-independent pathway by activating PLCβ signaling in T cells (93). This alternative pathway also triggers influx of Ca^2+^, activates PKC, ERK1/2, and ultimately nuclear translocation of NFκB and NF-AT.

Downstream of PI3K is the serine/threonine kinase Akt, which mediates many diverse biological processes upon specific binding to TCR, growth factors, insulin receptor, tyrosine kinase receptor, and GPCR. Two potent cytokines from superantigen-stimulated T cells, IFNγ and IL-2, also activate the PI3K/Akt/mTORC1 pathway *via* the transducer Janus kinase 1 (JAK1) after binding IFNγ and IL-2 receptors, respectively (94, 95). Site-specific phosphorylation/dephosphorylation modulates the PI3K/Akt signaling to activate mTORC1. Activation of mTORC1 causes phosphorylation and activation of the ribosomal 40S protein p70S6 kinase (p70S6K) plus eukaryotic initiation factor binding protein 1 (4EBP1) (96–101). Thus mTORC1 controls protein translation essential for G1 to S phase transition (100). An mTORC1-specific inhibitor, rapamycin, blocks SEB-induced T cell proliferation as well as SEB-induced IL-2 and IFNγ *in vitro* and *in vivo* (102). Importantly, inhibition of the SEB-induced PI3K/Akt/mTORC1 pathway by rapamycin prevents lethal toxic shock in a mouse model (102).

## Inflammatory Mediators, TCR/Costimulators, and MHC Class II Cross Linking Activate NFκB

*In vitro* and *in vivo* studies reveal that excessive release of proinflammatory cytokines (IL-1, TNFα, IL-6, and IFNγ) mediates the toxic effects of superantigens (49, 51, 52, 76, 79). Proinflammatory cytokines like IL-1 and TNFα activate the transcriptional factor NFκB in many cell types, thus perpetuating the inflammatory response. IL-1 and TNFα have tissue damaging effects, and together with SEB-induced metalloproteinases (MMP) and the pro-coagulant tissue factor (TF), promote inflammation and coagulation (87, 103). IL-2 from superantigen-activated T cells causes vasodilation, vascular leak, and edema.

Many cell types respond directly to staphylococcal superantigens besides T and APC/monocytes, including B, endothelial, synovial fibroblasts, intestinal epithelial, and mast (58, 79, 104–109). Cross-linking of TCR with MHC class II on B cells by superantigen triggers B cell proliferation and differentiation into plasma cells (104). Stimulation of synovial fibroblasts with superantigens induces chemokine gene expression, raising the possibility that superantigens can trigger chemotactic responses and initiate inflammatory arthritis (105). Human T84 colonic cells increase ion flow after incubation with SEB and PBMC, suggesting that superantigens indirectly affect gut mucosa *via* the immune system (106). SEB and TSST-1 transcytose intestinal epithelial cell barriers, unlike SEA (107), and TSST-1 can also induce chemotactic cytokines (IL-8 and MIP-3α) from human vaginal epithelial cells (109). *S. aureus* frequently colonizes vaginal mucosa, producing TSST-1, which penetrates the mucosa through chemokines from epithelial cells (110). Chemokines recruit neutrophils and other immune cells activated furthermore by superantigens. The potent activation of T cells by superantigen interaction of MHC class II and TCR induces T-cell proliferation *via* cell activation pathways in T cells and APC. The proliferative response as a result of IL-2 induction from superantigen-activated T cells is similar to that induced by mitogens. IL-2 increases vascular permeability, causing edema and multiple organ damage (111). TNFα synergizes with IL-2 to induce pulmonary vascular leak and lymphocyte accumulation (112). The combination of TNFα and IFNγ elaborated by SEB-activated cells disrupt the epithelial barrier, causing edema and vascular leak in gut mucosa (106).

Interleukin 1 interacts with IL-1 receptor 1 (IL-1R1) and an additional accessory protein, triggering downstream signaling molecules like the adaptor myeloid differentiation factor 88 (MyD88), IL-1R- associated protein kinase 1 (IRAK1), and TNF receptor-associated factor 6 (TRAF6) that activate NFκB (113). A set of structurally related receptors, the TLRs, signal with similar intracellular adaptors as those used for IL-1R1, but are not used for superantigen signaling (85, 114). The TLRs are conserved, type-1 transmembrane receptors that recognize pathogen-associated molecular patterns (PAMPs), such as lipoprotein, peptidoglycan, LPS, flagellin, dsRNA, and viral RNA, which stimulate host innate immunity and enhance adaptive immunity (85, 114). SEB reportedly increases cellular expression of TLR2 and TLR4, synergistically promoting lethal shock with LPS (115, 116). Activation of NFκB induces proinflammatory genes as well as pro- and anti-apoptotic genes. An auto-feedback loop exists to downregulate NFκB, which induces IκBα. Other cytosolic PRR sense intracellular damage-associated molecular patterns (DAMPs), triggering inflammasome activation and release of caspases plus IL-1β (117).

TNFα binds to TNF receptor 1 (TNFR1) or TNFR2, activating NFκB and inducing expression of other cytokines, adhesion, as well as co-stimulatory molecules (118, 119). The cytotoxic functions of TNFα are mostly mediated by binding TNFR1 *via* intracellular death domains that trigger apoptosis through caspase activation. SEB increases expression of CD95 (Fas), a receptor of the TNFR superfamily. Intracellular adaptors TNFR-associated death domain (TRADD), Fas-associated death domain (FADD), and receptor interacting protein kinase (RIP) are used by the TNFR superfamily to activate the caspase 8 cascade, JNK, and NFκB that subsequently elicit apoptosis, cell activation, coagulation, inflammation, and host defense (119). TNFα and IFNγ act synergistically on epithelial cells to increase ion transport, causing cell damage and epithelial leakage (120). The critical role of TNFα in mediating pathological effects of SEB and lethality was recognized early on as anti-TNFα antibodies confer protection from SEB-induced shock in a d-galactosamine (d-gal)-sensitized mouse model (121). Both IL-1 and TNFα enhance the procoagulant activity of TF and activate neutrophils, accounting for tissue damage and organ injury commonly seen in animal models of septic shock and SEB-induced lethal shock.

Type II interferon (IFNγ) binds to IFNγR, which belongs to the family of interferon receptors (IFNR), including the structurally different receptors for type I interferons (IFNα, IFNβ) (122, 123). The signal transducer and activator of transcription 1 (STAT1) is a common signal transducer for both types of IFNR, and phosphorylation of STAT1 by Janus kinases initiates signal transduction. IFNα and IFNβ induce indistinguishable signals, which include anti-proliferative and antiviral activities. Types I and II IFNs have many overlapping activities and stimulate many common interferon-stimulated genes (ISG) (123, 124). However, in addition to host-defense functions, IFNγ also induces immunoproteasomes and expression of MHC class II. IFNγ promotes cell-mediated immunological activities essential for antibacterial defense. The IFNγ-activated JAKs also activate PI3K in a STAT1-independent manner culminating in mTORC1 activation, promoting protein translation (125). IFNγ activates PKC leading to MAPK activation, which is also commonly activated by IL-1, TLR ligands, and TNFα. IFNγ has a critical role in host defense as the induction of immunoproteasome components and antigen-processing peptidases enhance cellular immune responses against pathogens. Both types of IFNs induce apoptosis and many ISGs have antiviral, anti-angiogenic, and immunomodulatory functions. IFNs induce apoptosis by activating death receptors such as Fas (CD95), which then activates the adaptor FADD, leading to subsequent caspase-8 activation. Activation of the caspase 8 cascade causes cell death plus release of cytochrome c and mitochondrial DNA (mtDNA). SEA-induced hepatotoxicity is mediated by FasL and the hepatocellular damage is independent of leukocyte recruitment (126). IFNγ disrupts barrier function and ion transport in superantigen-activated epithelial cells. *In vitro*, disturbances in epithelial barrier function can be duplicated with IFNγ plus TNFα (106). Recent studies suggest that IFNγ downregulates regulatory T-cells (Treg), accounting for the potent polarizing effects of IFNγ on TH1/TH2 cell differentiation and an inflammatory environment for cell migration and activation (127).

IL-2 binds to IL-2R and activates PI3K and Ras (128). Activation of the PI3K/Akt/mTORC1 pathway and Ras controls proliferation, growth, and differentiation of many cell types. Ras activates the MAPK and ERK cascades, leading to transcriptional activation of AP-1, cJun/Fos, and NFAT. The MAPK cascade induces ER stress and NFκB (129). IL-2 induces vasodilation and increases microvascular permeability by suppressing endothelin-1, causing perivascular edema seen in SEB-induced acute lung injury (130, 131). IL-2-deficient mice are resistant to SEB-induced toxic shock, providing evidence for the critical roles of PI3K/mTORC1 and NFκB (132).

The chemokines IL-8, MCP-1, MIP-1α, and MIP-1β are induced directly by SEB or TSST-1. These chemoattractant chemokines activate leukocytes and influence migration of neutrophils, dendritic cells, and leukocytes (133, 134). Chemokines bind to seven-transmembrane GPCR, induce early Ca^2+^ flux, activate PLC and signal *via* the PI3K/mTORC1 pathway (133). Cytokine- and chemokine-activated neutrophils, recruited to sites of tissue injury and inflammation, produce reactive oxygen species (ROS) and activate MMPs contributing to organ dysfunction. MMPs cause tissue degradation and change chemokine interactions with the extracellular matrix (ECM), creating a chemokine gradient affecting cell recruitment (133). Exudates from superantigen-injected air pouches are predominantly neutrophils with some macrophages (135). Both systemic and intranasal administration of SEB cause acute lung injury characterized by increased: (1) expression of adhesion molecules ICAM-1 and VCAM; (2) neutrophil and mononuclear cell infiltrate; (3) endothelial cell injury; and (4) vascular permeability (135–137).

The PI3K/mTORC1 pathway plays a dominant role in superantigen-induced cell proliferation and migration as TCR, CD28, IL-2R, IFNγR, and chemokine receptors all signal through this pathway. IL-1 and TNFα independently activate NFκB *via* MyD88/TRAF6/IRAK and FADD/TRADD/RIP, respectively (87). TCR and CD28 *via* PKC also activate NFκB signaling. Other PRR, including surface and cytosolic TLRs, signal *via* MyD88 to activate NFκB *via* different adaptors. In addition, TLR3 and TLR4 activate TRIF (toll/IL-1 receptor homology domain-containing adaptor inducing interferon β) signaling, inducing interferon regulatory factors (IRFs) (114). The overlap of PRR signaling by NFκB and IRFs with TCR and co-stimulatory signals by superantigens to activate NFκB and mTORC1 cannot be understated. These pathways activate inflammatory genes, as well as antiviral, anti-apoptotic, and pro-apoptotic molecules. Thus the three initial signals provided by TCR, costimulatory receptors, and cytokines converge on NFκB and mTORC1 to promote host defense against superantigens.

## *In Vivo* Effects of Superantigen

Injection of SEB into mice has been used to study activation-induced apoptosis and T-cell anergy *in vivo*. This effect may be linked to a rapid (within 1 h) loss of l-selectin on the surface of specific Vβ-bearing T cells, thus resulting in decreased signal transduction (138). *Via* endocytosis, surface levels of TCR-CD3 decrease ~50% among Vβ-reactive T cells within 30 min after SEB exposure (139). The rapid hyperactivation and proliferation of T cells in mice following an SEB injection is transient, as within 48 h the majority of proliferating T cells is eliminated by activation-induced cell death (140). CD95 mediates elimination of SEB-activated T cells and the residual Vβ-specific cells become anergic, or functionally unresponsive. However, controversy still exists regarding the functional ability and fate of these anergic T cells. After injection of SEB into mice, splenic Vβ8^+^ T cells are deleted or no longer respond to SEB, and produce less IL-2 and IFNγ. In contrast, others report that these anergic cells synthesize less IL-2 but still secrete IFNγ that can mediate toxic shock following a subsequent dose of SEB (141). An evident paradox is that the anti-inflammatory cytokine IL-10, which protects against SE-induced shock (142), is also produced by SEB-primed T cells. Treg cells likely downregulate superantigen activation *in vitro* and *in vivo*.

Immune homeostasis is maintained by Treg cells through a number of mechanisms depending on the “immune environment” (143). Natural and induced Foxp3^+^ Treg cells control excessive immune activation deleterious to the host. Treg cells also downregulate autoimmune responses, and superantigens can subvert the functional activity of Treg cells in atopic dermatitis (144). Although the immunosuppressive function of Treg cells is well known, the mechanisms of action and involvement of different cell types are still debated. The markers used in identifying different Treg cells are evolving, but the surface receptor cytotoxic T lymphocyte antigen 4 (CTLA4) likely plays a role particularly in superantigen-related counter regulation (145). CTLA4 interacts with costimulatory molecules CD80 and CD86, preventing costimulatory signals in superantigen-activated T cells (146). Systemic inflammatory responses elicited by SEB actually destabilize Treg cells and additional expansion of them *in vivo* does not protect transgenic mice from SEB-induced toxic shock (147).

## Superantigen-Induced Autoimmunity

The ability of superantigens to cross-link MHC class II and specific TCR Vβ enables these microbial toxins to stimulate the immune system and induce autoimmunity by activating APC and normally quiescent, autoreactive T- and B-cells. Activation results in cytokine and chemokine release, thus mediating a potently acute inflammatory response. Several experimental animal models show that staphylococcal superantigens are arthrogenic (148, 149). TSST-1 exacerbates bacterial cell wall-induced arthritis in rats, possibly linked to accumulating Vβ11^+^ T cells and IFNγ production within arthritic joints (149). TSST-1 also plays a pivotal role in murine septic arthritis, as the frequency and severity of this disease are increased after intravenous administration of TSST-1-secreting *S. aureus* (150). SEA or SEB can also induce relapses of experimental autoimmune encephalomyelitis in a murine model for multiple sclerosis (151). How exogenously administered toxin triggers autoimmune processes like arthritis is unknown, but it is likely that proinflammatory cytokines and chemokines produced in response to superantigens facilitate specific recruitment and migration of autoreactive T-cells into synovial tissue and joints. Thus, proinflammatory cytokines and chemokines have acute toxic effects promoting cell activation and recruitment. Following minor tissue injury or inflammation due to superantigen exposure, an increased presence of immune cells might initiate a destructive autoimmune reaction.

Psoriasis and atopic dermatitis also represent autoimmune diseases linked to staphylococcal and streptococcal colonization of skin and subsequent production of superantigens (152). SEB on healthy human skin induces inflammatory reactions, possibly linked to degranulation of cutaneous mast cells (58). T cells from patients with severe atopic dermatitis are apoptotic, which may lead to chronic infections and subsequent worsening of disease (153). Bacterial density on skin can affect relative sensitivity toward these toxins and development of atopic dermatitis.

## Pathway Analysis Yields Potential Drug Targets

There are currently no available therapeutics for treating superantigen-induced shock, except for the use of intravenous human immunoglobulin (154, 155). Targeting and neutralizing a superantigen directly is most suitable at early stages of exposure before cell activation and initiation of the proinflammatory cytokine cascade. Therapies targeting superantigens at the receptor level have been extensively reviewed recently (156) and include receptor-blocking peptides derived from toxins, chimeric inhibitors composed of Vβ and MHC class II domains, as well as synthetic blockers of co-stimulatory receptor CD28. However, preventing toxin-receptor interaction is ineffective post-toxin exposure and some inhibitors must be tailored to target individual toxins. Failed sepsis clinical trials of eritoran (anti-endotoxin), a drug that prevents the early steps of receptor interaction, suggest that blocking superantigen–receptor interactions will likely not protect against SEB-induced shock (157).

An important class of therapeutic compounds blocks signal transduction pathways activated by superantigens, and as these events are post-exposure, they are perhaps more amenable to suppressive manipulation. One example is the NFκB cascade containing many upstream activators. *In vitro* studies indicate numerous genes for cell adhesion molecules, cytokines, chemokines, acute phase proteins, and inducible nitric oxide synthase that are implicated in superantigen-induced lethal shock and contain NFκB binding sites in the promotor/enhancer region (84). Activation of NFĸB leads to the inducible expression of many mediators involved in inflammation and tissue injury seen in SEB-induced lung injury and toxic shock models. Inhibition of NFκB is beneficial, or has no effect, in preventing SEB-induced shock depending upon the mouse model (136, 158, 159); however, NFκB inhibitor must be given early and for a long duration to afford any protection (136).

Pathway inhibitors are used for identifying molecules and signaling pathways crucial for cellular responses to a specific stimulus. The superantigenic properties of SEB make it an “ideal” toxin to study cell activation signals and molecular pathways. An obvious step in testing new therapeutic approaches for SEB-induced shock is finding relevant animal models that mimic important aspects of human disease. Mice are much less susceptible to SEB due to lower affinity of superantigen for mouse MHC class II (51, 62, 160). Potentiating agents such as LPS, viruses, d-gal, and actinomycin D are used together with SEB to sensitize mice (50–52). These synergistic agents alone activate similar host signaling pathways as superantigens, confounding the “pure” host response to SEB. An alternative model utilizes transgenic mice, with either human HLA-DR3 or -DQ8, that lethally respond to SEs without a potentiating agent (161, 162). Another recent, simplified model employs “double-hit,” low dose SEB in C3H/HeJ mice, an LPS-resistant strain (131). Pathological lesions, cytokine response, and time to lethality in this “double-hit” model resemble findings in non-human primates (NHP) and staphylococcal toxic shock syndrome in patients (49). Host signatures arising from “SEB-only” exposure unexpectedly include many IFN-induced genes in multiple organs, not previously linked to SEB pathogenesis (163).

## Oligonucleotide Microarray Reveals Seb-Induced Danger Signals

Oligonucleotide microarray analysis in the “double-hit” SEB mouse model reveals induction of danger signals bearing IFN signatures (163). These genes cover five important molecular hubs signaling danger similar to those activated by IFNs and pathogens. The upregulated transcripts present in PBMC, spleen, lung, liver, kidney, and heart include (1) innate mediators; (2) DNA/RNA sensing system; (3) ER stress; (4) metabolic/oxidative stress; and (5) the apoptosis pathway. Proinflammatory cytokines IL-1, IL-6, TNFα, IFNγ, and IFNγ-induced chemokines (CXCL9, CXCL10, CXCL11) are most prominent in SEB-stimulated PBMC, confirming previous observations *in vitro* and *in vivo* (51, 86, 135, 161, 162, 164). Activation of other innate host-defense genes include the Fc receptor for phagocytosis, MHC class I and II for increased APC function, as well as cell-surface receptors and adhesion molecules to promote cell recruitment. Z-DNA binding protein 1 (ZBP1), a DNA sensor triggering ISGs *via* DNA binding (165), is surprisingly upregulated in all tissues from the “double-hit” SEB mouse model (163). T-cell proliferation requires elevated protein translation and increased metabolism, resulting in misfolded proteins and oxidative stress. ER stress response genes such as ubiquitin ligases, immunoproteasome components, proteasome peptidases, and WARS (tryptophanyl-tRNA synthetase) are likely a result of Ca^2+^ flux, unfolded proteins, and activated PKC following cell activation and increased protein synthesis. Unresolved ER stress activates caspases and apoptosis (166, 167). Enhanced activity of the mitochondrial electron transport chain ultimately leads to oxidative stress, evidenced by activated NADPH oxidase in lung (163). MMP, cathepsins, and other proteases from lysosomes are also seen in this mouse model and *in vitro* stimulation of PBMC with SEA or SEB (168, 169). Increased ROS and protease levels are major factors in organ injury. Although the “double-hit” mouse model is not perfect, it mimics many *in vivo* responses of NHP to SEB and there are many similarities of gene expression by mouse and human PBMC incubated with SEB.

The cell death pathway triggered *in vitro* and *in vivo* includes genes associated with apoptosis such as FADD, death receptor ligand TRAIL, caspases, CARD, and phospholipid scramblase 1 (PLSCR1). These genes are activated in PBMC and major organs from the “double-hit” SEB model and human PBMC, following SEA or SEB exposure (163, 168, 169). PLSCR1 is implicated in moving phosphatidyl serine to the outside plasma membrane of apoptotic cells. Other danger signals include K^+^ efflux, particulate ligands such as cholesterol crystals and lysosome destabilization that triggers inflammasome activation *via* NLRP3 which converts pro-IL-1β to IL-1β through caspase 1 (117). Catabolic enzymes cause destruction of cell matrix, accounting for the liver and lung injury seen in SEB-mediated shock.

Induction of ZBP1 leads to binding of DNA and endosomal TLR9, upregulating antiviral genes *via* the IRF3 and IRF7 (114, 170). TLR9 activation also promotes NFκB-mediated cytokine gene transcription and inflammasome activation (171). Mitochondria are the most likely source of cytosolic DNA induced by SEB, as ER stress and increased mitochondrial respiration due to T-cell proliferation and activation are known to induce ROS (172). Elevated ROS activates the intrinsic cell death pathway *via* caspase 9, leading to mitochondrial damage that releases cytochrome c and mtDNA (173). MtDNA acts as a direct inducer of ZBP1. In addition, mtDNA has motifs similar to bacterial DNA (CpG), binds cytosolic TLR9, and promotes activation of NFκB and IRFs. Mitochondrial ROS is also a potent inducer of the inflammasome NLRP3 (117). Both NLRP3 and CARD are identified in microarray studies *in vivo* and *in vitro* with superantigen-stimulated PBMC. Cathepsins, another category of “destroyer” molecules identified by microarray in SEB-activated PBMC (169), are indicative of lysosomal rupture which also activates inflammasomes.

Upon comparing the microarray data from an *in vivo* “SEB-only” mouse model (163), and human PBMC stimulated with SEA (168) or SEB (169), several commonly activated genes importantly induce the pathogenic effects of SEB (Table 2). These genes are activated by IFN and account for the therapeutic effectiveness of rapamycin in preventing SEB-induced shock (102).

**Table 2:** **Common differentially expressed genes induced by superantigens *in vitro* and *in vivo***.

Pathway/network	Gene	Major function
Innate response	IL6, TNFα, LTA, IL17A, IL22	Host defense, inflammation
	IFNγ	Host defense, antimicrobial
	CXCL11, CXXC5, CCL7, XCL1	Host defense, cell migration
	CISH, CIITA, GBP2, TRAF1, RGS16	Signal transduction
	PDE4DIP, PDE4B, PTGER3, P2RY14	Signal transduction
	NEDD9, GNAS, CSF1R	Signal transduction
	STAT1, STAT2, STAT3, IRF7	Transcription factor (TF)
	BATF, BATF2	IFN-inducible TF
	SOCS1, SOCS2, SOCS3	JAK/STAT counter-regulator
	CD69, CD74, ICAM	Immune regulation
	NRP2	Vascular signaling
	Rel A, Rel, NFκBia	NFκB regulator
DNA damage response	RIPK2	DNA sensor interactor
	CTPS, UPP1	Nucleic acid synthesis
	PIM1, PIM2	DNA repair/assembly
	GADD45G	DNA repair adaptor
ER stress/oxidative stress	SIAH2	Ubiquitin E3 ligase
	KCNE4	Membrane integrity
	JunB	Stress response TF
	MGST1	Cell protection
Metabolic stress	IL2, IL2RA, MACF1	Cell proliferation regulator
	FABP4, CD36	Fatty acid metabolism
	HK1, PDK4, PGS1	Cell metabolism
	TARS, NDST2	Synthetase
Apoptosis	PLSCR1, NR4A1	Membrane integrity
	CD40, TNFRSF9	TNFRSF, death receptor
	Casp 4, CFLAR	Caspase regulator
	VCAN, LMNB1	Cell matrix breakdown
	BCL2, BCL6	Anti-apoptotic regulator
	CCND2	Cell cycle regulator
	PLA2G7	Cardiovascular damage
Others	ARID5A, ZBTB32, NDST2	

*ARID5A, AT-rich interactive domain 5A (MRF1-like); BATF2, basic leucine zipper transcription factor (TF); CCND2, cAMP specific cyclin D2; CFLAR, caspase 8 and FADD-like apoptosis regulator; CIITA, MHC class II transactivator; CISH, cytokine inducible SH2-containg protein; CTPS, cytidine 5′-triphosphate synthase; FABP4, fatty acid binding protein 4; GADD45G, growth arrest and DNA-damage-inducible 45 gamma; GBP2, IFN-inducible guanylate binding protein 2; GNAS, guanine nucleotide binding protein; HK1, hexokinase 1; KCNE3, potassium voltage-gated channel; LMNB1, lamin B1; MACF1, microtubule-actin crosslinking factor 1; MGST1, microsomal glutathione-*S*-transferase 1; NDST2, *N*-deacetylase/*N*-sulfotransferase; NEDD9 (HEF1), neural precursor cell expressed; NR4A1, nuclear receptor subfamily 4, group A, membrane 1; NRP2, neuropilin transmembrane protein receptor; P2RY14, purinergic receptor P2Y, G protein coupled; PDE4DIP, phosphodiesterase 4D interacting protein; PDE4B, cAMP specific phosphodiesterase 4B; PDK4, pyruvate dehydrogenase kinase isoenzyme 4; PGS1, phosphatidylglycerophosphate synthase 1; PIM1, pro-viral DNA integration; PLA2G7, phospholipase A2 group VII; PLSCR1, phospholipid scramblase; PIM1, proviral integration site 1; PTGER3, prostaglandin E receptor 3; RGS16, regulator of G protein signaling 16; RIPK2 (RIP2), TNFRSF-interacting protein kinase; SIAH2, seven *in absentia* 2; SLC30A1, solute carrier family 30 (zinc transporter); SOCS1, suppressor of cytokine signaling 1; TARS, threonyl-tRNA synthetase; UPP1, uridine phosphorylase 1; VCAN, verican; ZBTB32, zinc finger and BTB domain containing 32*.

## Drug Targets: What Works and What Does Not

The early induction of three key proinflammatory cytokines, IL-1, TNFα, and IFNγ, work *via* individual receptor-mediated signaling molecules that, respectively, activate distinct pathways: (1) IL-1R/MyD88/NFκB; (2) TNFαR/FADD/RIP; and (3) IFNγR/JAK/IRF. IL-1, TNFα, and IFNγ have independent and synergistic effects signaling inflammation, caspase activation/cell death, and an antiviral response. IL-1 from inflammasome activation has pleiotropic effects, while TNFα has an established role that initiates cell death through the adaptor FADD, activating caspases 3 and 8. IFNγ triggers innate host defense responses, antiviral genes, apoptotic programs, immunoproteasomes, and has many immunomodulatory functions. Interruption of these concurrent cascades early after SEB exposure is effective in preventing SEB-induced lethal shock.

Decades of drug development against sepsis and septic shock point to the failure of using anti-inflammatory cytokines alone, and early interruption of cytokine release is perhaps a necessary but insufficient target. Drugs also act on multiple targets, some known and unknown, as exemplified by statins that inhibit 3-hydroxy-3-methylglutaryl-coenzyme A with anti-inflammatory effects (174). Statins, used therapeutically to reduce cholesterol, are under consideration for treating various inflammatory diseases (175). Superantigens trigger multiple pathways that cross-regulate each other positively and negatively, thus targeting downstream effectors might be more specific and perhaps interfere less with normal cell function. Knowledge of immunoregulation within activated pathways by SEB enables a better choice of inhibitors.

Apoptosis plays a critical role in sepsis-induced lethality (176). The two pathways leading to apoptosis are operative in SEB-induced lethal shock, as induction of genes for both pathways occurs in the “double-hit” SEB model and other mouse models employing potentiating agents such as d-gal. The death receptor pathway used by the TNFR superfamily with ligands like TNFα and FasL induces cell death following superantigen exposure (121, 126). A second apoptosis pathway, the intrinsic pathway, is dependent on mitochondria and the Bcl2 family of pro- and anti-apoptotic proteins. ROS and loss of mitochondrial transmembrane potential play important roles, as previous studies implicate both in SEB pathogenesis (177). Other cell-surface receptors like CD44 might also contribute to SEB-induced injury, as CD44-knockout mice have elevated liver damage in the d-gal-sensitized SEB model (178). Since d-gal is hepatotoxic, the induction of pro-apoptotic molecules by SEB likely act synergistically with d-gal to promote cell death.

Microarray gene analysis in an “SEB-only” mouse model implicates both extrinsic and intrinsic pathways in SEB-induced apoptosis. Apoptosis plays a role in down-regulating immune responses but simultaneously has devastating effects when apoptotic cells or associated molecules are not removed. Autophagy is a cellular mechanism that removes bacteria, protein aggregates, and damaged organelles to maintain homeostasis (176, 179). A recent study indicates that blocking autophagy augments T-cell activation (180). Degradation and removal of Bcl10, which is part of CBM and a critical component for TCR and costimulatory signaling, is dependent on autophagy (176). IFN induces many genes regulating NFκB and apoptosis. The damage response induced by superantigens likely starts with inflammatory cytokines and apoptotic programs activated by IFNγ and TNFα. DAMPs such as mitochondrial ROS and mtDNA trigger more apoptosis, activate inflammasomes, and induce transcription factors for ISGs. Increasing energy demand and mitochondrial respiratory-chain activity also lead to elevated ROS. Normally, mitochondria damaged by excessive membrane permeability and ROS are removed by a specialized form of autophagy called mitophagy. However, overactivation of PI3K/mTORC1 in superantigen-stimulated cells likely blocks autophagy, resulting in inflammasome activation and accumulation of damaged mitochondria. Rapamycin, a well-known inducer of autophagy, prevents SEB-induced shock by removing damage-inducing molecules and damaged mitochondria. Two other FDA-approved immunosuppressants for organ transplants, cyclosporine A and tacrolimus, do not protect against superantigen-induced shock in NHP and human HLA-DR3 transgenic mice, respectively (181, 182). The calcineurin inhibitor, cyclosporine A, protects d-gal-sensitized mice from SEB-induced shock (121) but does not protect NHP challenged with SEB (181). Tacrolimus suppresses SEB-induced T-cell proliferation *in vitro* but does not confer protection from toxic shock in transgenic mice (182). Tacrolimus also fails to protect mice from lethal pneumonia induced by superantigen-producing *S. aureus* (182).

There is good agreement between the genes significantly induced in mouse PBMC from this “double-hit” SEB model and SEA- or SEG-stimulated human PBMC (163, 168). Many genes of the apoptosis-related cell death pathway account for the damage response initiated by SEB. MtDNA is ancestrally related to bacterial DNA (CpG motifs), inducing a “foreign” DNA sensor (ZBP1) or alternatively binding endosomal TLR9 that triggers host defense including type 1 IFN-mediated responses *via* IRF3 (114). Important clues from animal models, old and new, reveal acute release of proinflammatory cytokines that culminate in damaged organs and lethal shock.

## Conclusion

*Staphylococcus aureus* is a toxin-producing pathogen that causes various diseases found throughout the body. Increasingly, *S. aureus* becomes more resistant to various therapeutics (i.e., antibiotics) over time, thus our own immune systems must more effectively clear this pathogen. Further knowledge of our immune system will clearly enable us to better thwart *S. aureus* and other pathogens. Mammals sense invading microbes *via* conserved PRR for detecting molecular patterns on, or released by, various bacterial, viral, and fungal pathogens. This rapid innate immune response produces proinflammatory mediators, cell activation, and recruitment of inflammatory cells to infection sites. However, some sensors for detecting PAMPs also bind to host DAMPs, confusing the “stranger” versus “danger” signaling. Host response to staphylococcal superantigens typifies the generation of these danger signals as shown in a mouse model of SEB-induced shock. The induction of cell death through apoptotic proteins observed during sepsis or superantigen exposure may provide a common target for therapeutic intervention.

## Author Contributions

All authors listed have made substantial, direct and intellectual contributions to the work, and approved it for publication.

## Disclaimer

The views expressed in this publication are those of the authors and do not reflect the official policy or position of the Department of the Army, the Department of Defense, or the U.S. Government.

## Conflict of Interest Statement

The authors declare that the research was conducted in the absence of any commercial or financial relationships that could be construed as a potential conflict of interest.
